# Systematic engineering design helps creating new soft machines

**DOI:** 10.1186/s40638-018-0088-4

**Published:** 2018-10-26

**Authors:** Arthur Seibel, Lars Schiller

**Affiliations:** Workgroup on System Technologies and Engineering Design Methodology, Hamburg University of Technology, 21073 Hamburg, Germany

**Keywords:** Soft robotics, Design methodology, Gecko-inspired robot, Climbing robot

## Abstract

Soft robotics is an emerging field in the robotics community which deals with completely new types of robots. However, often new soft robotic designs depend on the ingenuity of the engineer rather being systematically derived. For this reason, in order to support the engineer in the design process, we present a design methodology for general technical systems in this paper and explain it in depth in the context of soft robotics. The design methodology consists of a combination of state-of-the-art engineering concepts that are arranged in such a way that the engineer is guided through the design process. The effectiveness of a systematic approach in soft robotics is illustrated on the design of a new gecko-inspired, climbing soft robot.

**Electronic supplementary material:**

The online version of this article (10.1186/s40638-018-0088-4) contains supplementary material, which is available to authorized users.

## Background

Soft robotics is a fast-growing field in the robotics sciences. This rather young discipline deals with robots made entirely of soft materials (such as silicones) or materials with soft behavior (such as granules). Often, but not necessarily, the design of a soft robot is biologically inspired. Examples are the reproduction of the tail of a fish [1] or the tentacle of an octopus [2].

Due to their flexibility, soft robots have many advantages over conventional, hard robots. For example, deformable structures play an important role in applications with high uncertainty, such as movement in impassable and unknown environments [3, 4] or gripping of unknown objects [5–8]. The softness also allows a safe contact with living organisms without a potential risk of injuries [6]. In addition, deformable structures are able to store and release energy—a beneficial property for energy-efficient movement [9].

Typically, in the soft robotics literature, only the realization of the introduced system is presented, but the concrete path to this solution is not further specified, leaving the designer of new soft machines without proper guidance. For this reason, we introduce a general design methodology for technical systems in this paper and describe it in detail in the context of soft robotics. The methodology consists of several basic engineering concepts that are structured to guide the engineer through the design process. The effectiveness of this methodology in creating new solutions in soft robotics is demonstrated on the design of a climbing soft robot inspired by the gecko.

## Methods

**Fig. 1:**
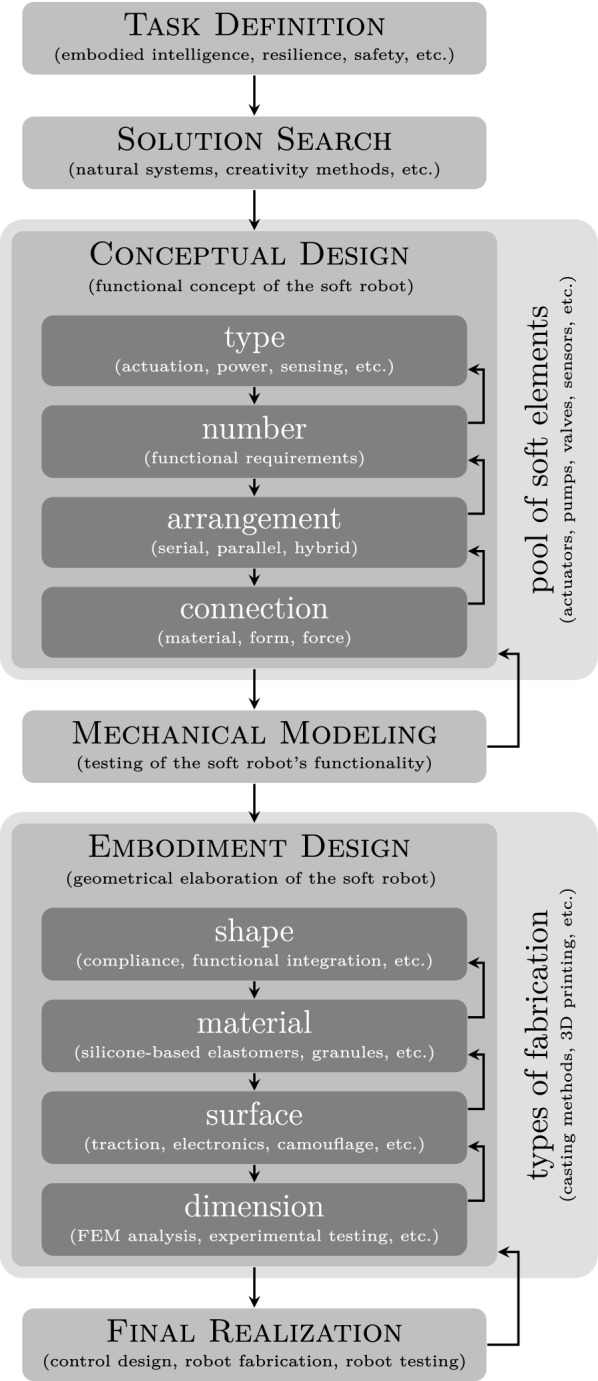
General design methodology for technical systems in the context of soft robotics

The proposed design methodology is illustrated in Fig. 1. The design process starts with defining the *task*, followed by searching for a suitable *solution*. Then, based on this solution, the *conceptual design* of the soft robot is carried out, whose functionality is examined by a mechanical *model*. Afterward, the functional concept is elaborated in the *embodiment design* stage, and the design process finally ends with the *realization* of the robot. As indicated in the figure, this process is iterative, in which steps can be merged, omitted, and skipped.

**Fig. 2:**
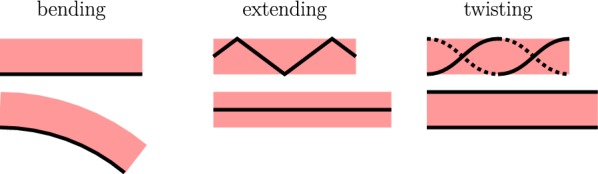
Types of soft actuators in resting (top) and actuated states (bottom). Red: stretchable part, black: non-stretchable part(s)

**Fig. 3:**
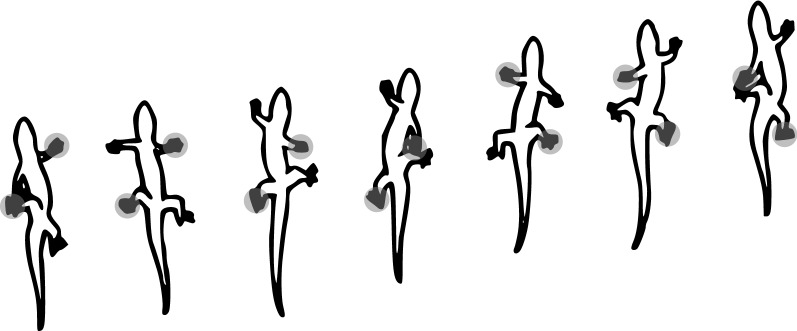
Gait pattern of the gecko during wall climbing (figure adapted from [58]). Gray circles represent feet attachment

### Task definition

In the broad engineering sense, a task means recognizing a problem or need *and* translating it into a technical goal. A typical task that can be well addressed within the framework of soft robotics is the design of a technical system whose intelligence is not located outside the body but is integrated into the structure itself, also known as embodied intelligence [10]. This property reduces the need for complicated sensor systems and feedback controllers and provides a significant advantage over hard robotics. Another typical task that can be well solved in a soft way is to create a robot that does not harm its environment and is also not harmed by external influences.

### Solution search

A typical way for finding new solutions in soft robotics is by means of analysis of natural systems [11]. An important part of such an approach is finding a sufficient level of abstraction of the underlying principles and transferring them into a robot system by using state-of-the-art technology [12]. Other possible ways for finding suitable solutions for new soft robotic designs are by transferring already existing technical systems into soft counterparts or by using creativity methods [13].

**Fig. 4:**
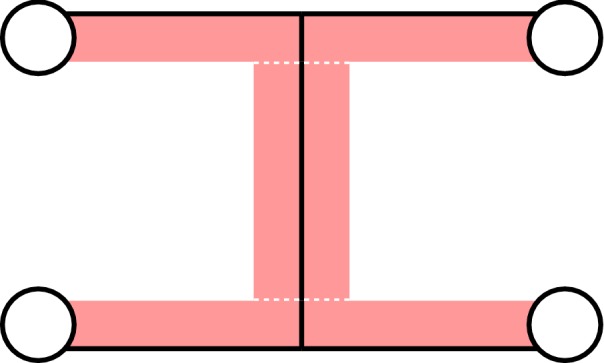
Arrangement of bending actuators (red) and suction cups (white) to form our soft robot

**Fig. 5:**
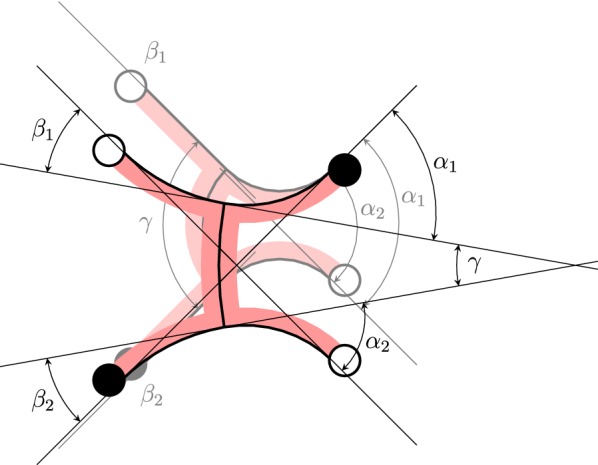
Mechanical model of our soft robot in a slightly ($\gamma =20^\circ$, front) and a fully actuated state ($\gamma =90^\circ$, back). Filled circles represent attached feet

**Fig. 6:**
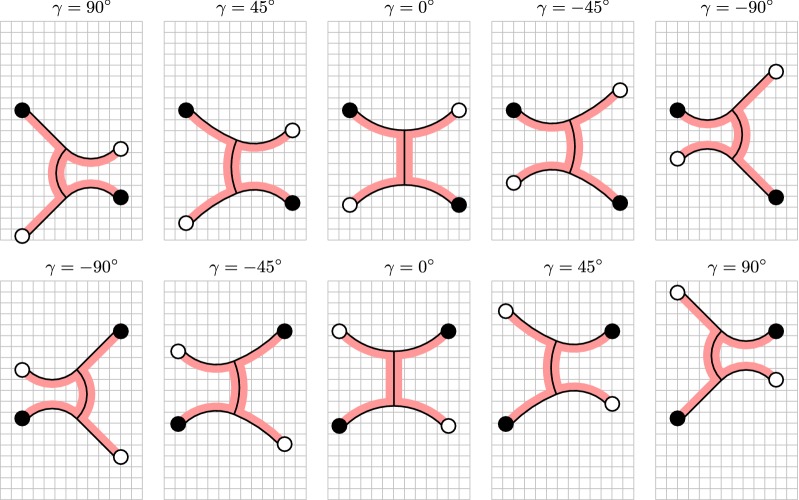
Gait pattern of our soft robot. Filled circles represent feet attachment

**Fig. 7:**
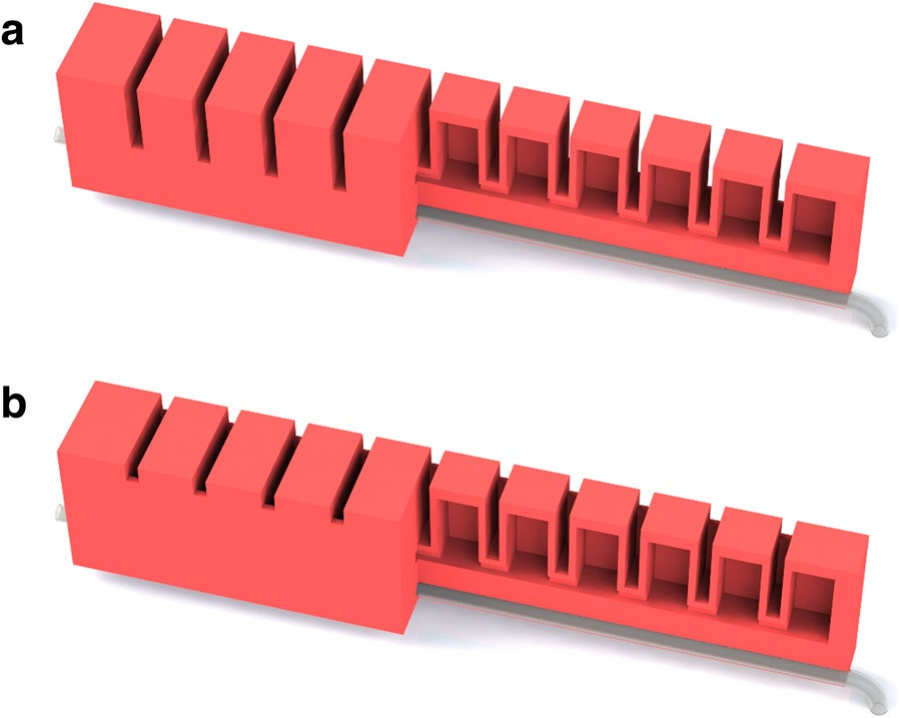
Partially cut CAD models of the bending actuator designs used for the torso (**a**) and the legs (**b**) of our soft robot. **a** Actuator design without side walls, **b** actuator design with side walls (the wall thickness is 1 mm)

### Conceptual design

A technical system can generally be described by the *type*, *number*, *arrangement*, and *connection* of elements, cf. Fig. 1. Here, the term “element” refers to a part of the system that fulfills a certain function. A function, in turn, is realized by a suitable working principle [13]. The result of this design stage is a concept of the soft robot, whose focus lies on its functionality.

#### Elements

Typical elements that are important for soft robotics applications are soft actuators. Basically, a soft actuator consists of a stretchable part and one (or more) non-stretchable (but bendable) part(s); see Fig. 2. Depending on the function, the non-stretchable part of the soft actuator can be arranged as follows. For bending, it is placed on the outer surface of the actuator (Fig. 2, left). For extending, it is integrated into the actuator, for example, in a zigzag manner (Fig. 2, center). And for twisting, it is wrapped around the actuator (Fig. 2, right). The actuation is realized, for example, by using length-variable tendons or by dividing the stretchable part into one (or more) inflatable chamber(s) [14]. Furthermore, other principles for soft actuators exist to perform, for example, curling [6, 15], contracting [16, 17], rotating [18–20], or other complex motions [21, 22].

Other important elements in soft robotics are suction cups. Three types of suction cups can be currently found in the literature: suction cups that are actuated by a dielectric elastomer [23], suction cups that are actuated by negative [24], and suction cups that are actuated by positive air pressure [25]. The latter type is based on the pneu-net principle from [6].

The pool of elements may also include soft pumps [26–30], soft valves [31–33], soft sensors [14, 34], and other possible soft devices.

#### Type

According to the defined task, in this step, we have to select the required types of elements from the pool summarized above. For production reasons, as many identical parts as possible should be used.

#### Number

The number of elements in technical systems basically depends on the functional requirements of the system to be designed. Ideally, as few parts as possible should be used, that is, an integral design is to be preferred.

**Fig. 8:**
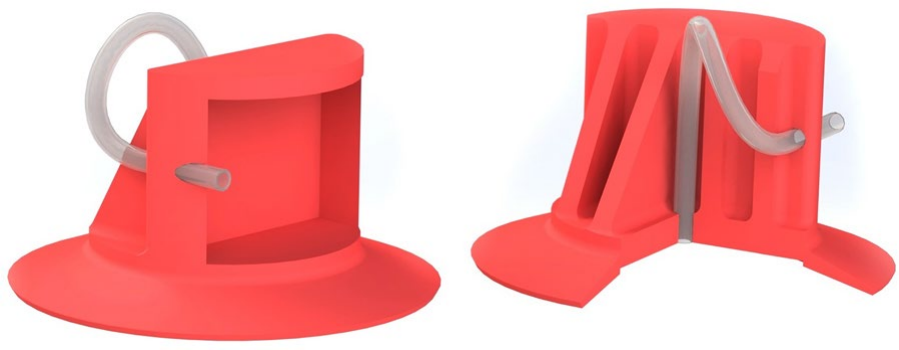
Partially cut CAD model of the suction cup design used in our soft robot in two different views

**Fig. 9:**
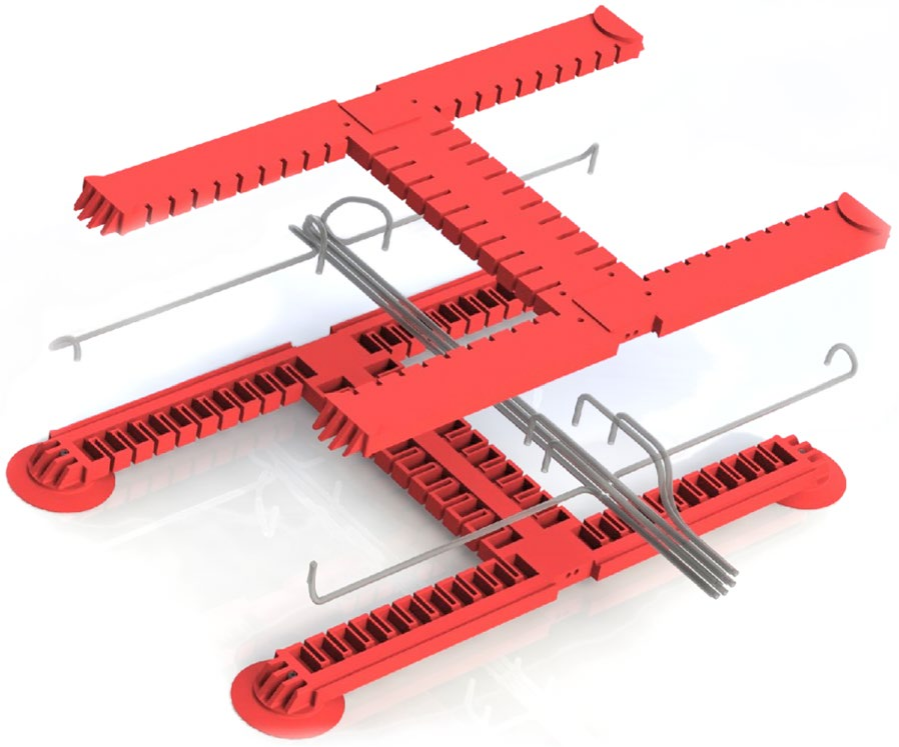
Horizontally cut, exploded CAD model of the embodiment design of our soft robot

**Fig. 10:**
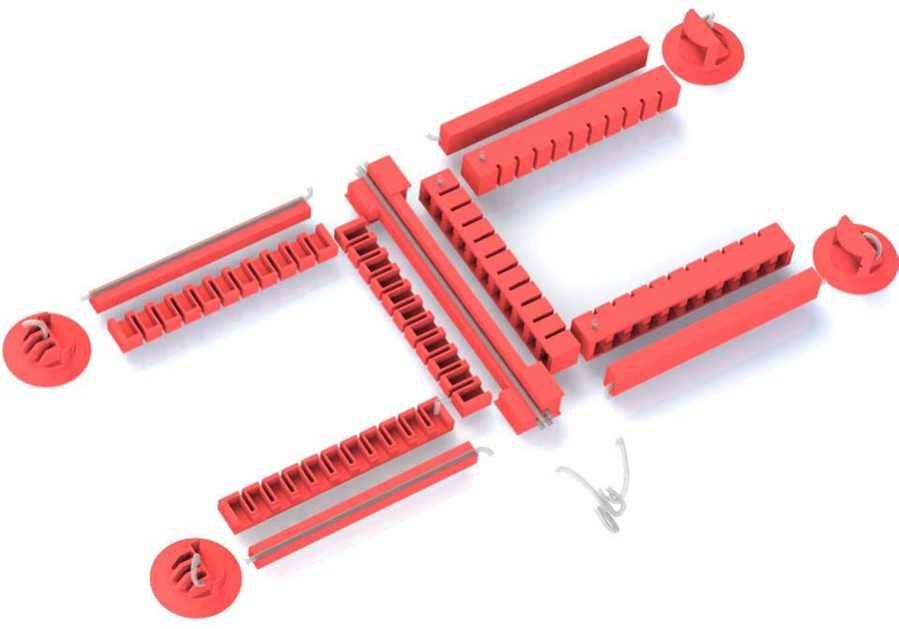
Partially cut, exploded CAD model of the individual parts of our soft robot

#### Arrangement

The arrangement of elements in a technical system can basically be realized in *series*, in *parallel*, or in *combination* of both. Examples of serial, parallel, and hybrid arrangement of bending actuators are given in [35, 36].

**Fig. 11:**
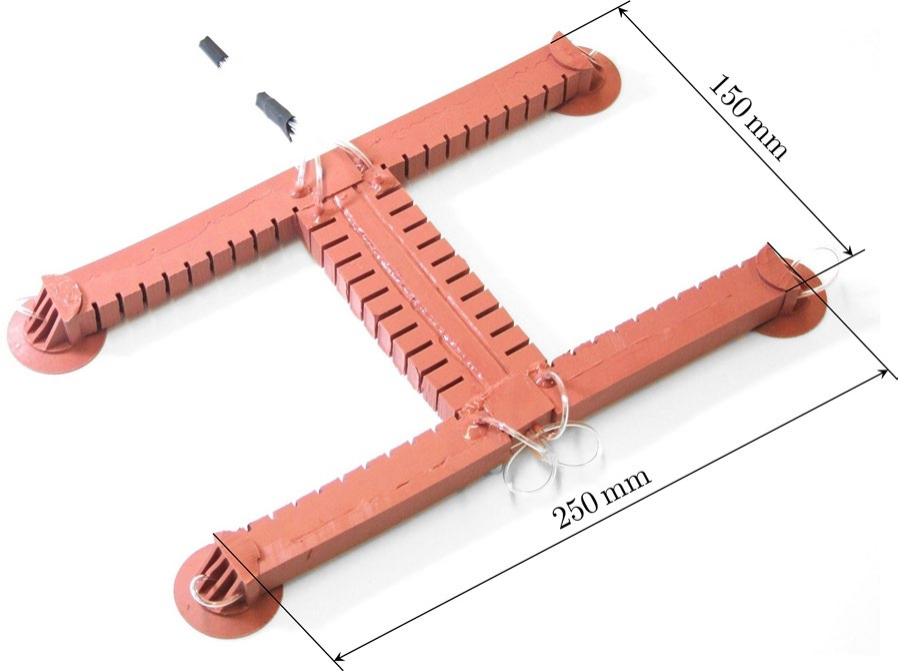
Photograph of our fabricated soft robot. The height of the robot is 20 mm and the weight is 200 g

**Fig. 12:**
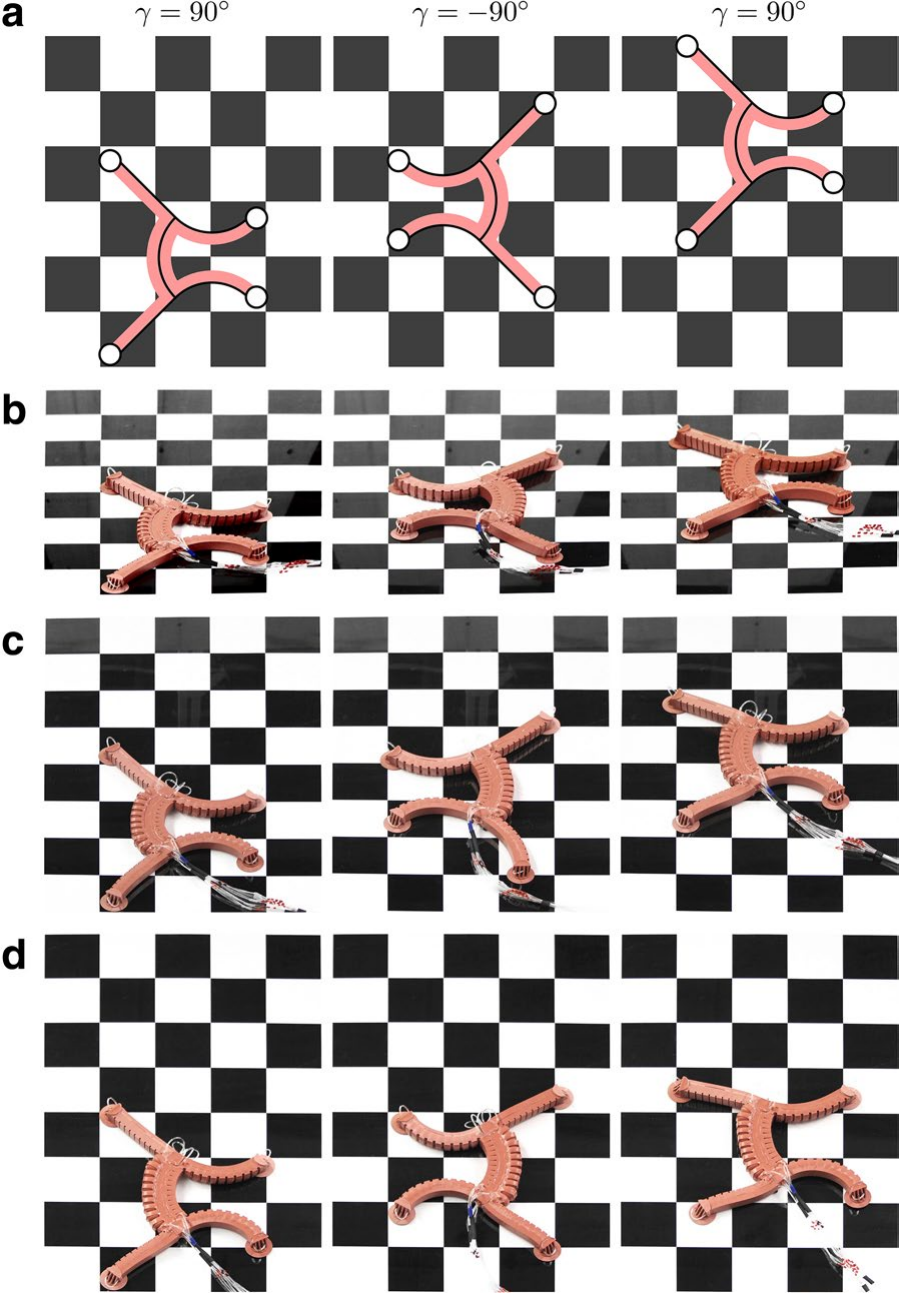
Gait performance of our soft robot for different inclination angles $\delta$ in comparison with the simulation. **a** Simulation, **b** $\delta =0^\circ$, **c** $\delta =20^\circ$, **d** $\delta =40^\circ$. The box width is 8 cm

#### Connection

The connection of elements in technical systems can basically be achieved by *material*, *form*, or *force*. A typical material connection in soft robotics is by gluing the elements together [36]. Form connection can be realized, for example, by dovetail joints [37] and force connection, for example, by friction fit via click-bricks [38] or by integrated magnets [39].

### Mechanical modeling

In order to analyze whether the concept from the previous design stage fulfills the required functionality, it is recommended to develop a (simplified) mechanical model of the soft robot. This model may later form the basis for the control design in the final realization stage of the design process. Helpful concepts here are the piecewise constant curvature assumption and beam theory [40].

### Embodiment design

In this design stage, we define the *shape*, *material*, *surface*, and *dimension* of the solution concept from the conceptual design stage. Furthermore, also the fabrication method is specified. The result of this design stage is the geometrical elaboration of the soft robot. In this context, the Soft Robotics Toolkit [41] provides a detailed collection of the embodiment designs of different elements.

#### Shape

The shape of an element or the entire technical system is understood as its geometrical form taking into account various constraints (like functional, manufacturing, esthetic). A typical shape of a soft robot exhibits a compliance similar to that of living organisms [42]. The goal here is to achieve a maximum functional integration into one body.

#### Material

The material in technical systems is typically selected according to functional, manufacturing, and economical requirements. A small overview of (silicone-based) elastomeric materials for soft robotics applications is given in Table 1. Ecoflex and Elastosil are highly extensible under low stresses and are typically used for the elastic parts of a soft robot. In contrast, PDMS is less deformable and is best suited for the more rigid parts of soft machines. All listed materials are two-component, silicone-based elastomers that exhibit a hyperelastic and viscoelastic behavior. They are resistant to mechanical damage [43] and can withstand fire, water, and snow [4].

**Table 1:** Typical (silicone-based) elastomeric materials used in soft robotics applications

	Ecoflex		Elastosil	Sylgard (PDMS)
Manufacturer	Smooth-on		Wacker Chemie	Dow Corning
Type	00-30	00-50	M 4601	184
Color	Translucent		Reddish brown	Transparent
Shore hardness	00-30	00-50	28 A	50 A
Tensile strength	1.38 MPa	2.17 MPa	6.50 MPa	6.76 MPa
Elongation at break	900%	980%	700%	150%

The listed information is taken from the data sheets of the suppliers

Further material types that are typically used in soft robotics are, for example, electroactive polymers [44], hydrogels [45], granules [5, 46, 47], fibers [48, 49], fabrics [4, 48, 50], and paper [16, 51].

#### Surface

The surface is an important but often overlooked aspect of a technical system. For example, the traction of a soft robot can be improved by introducing a texture on the contact surfaces [6, 15]. Furthermore, flexible and stretchable electronics [52] can be placed on the surfaces of a soft machine. And even the use for camouflage and display is reported in the literature [53]. So, by utilizing the *free* surfaces of a soft robot, additional functions can be integrated into the system.

#### Dimension

The dimensions of technical systems basically depend on the desired shape, functional requirements, and permissible material stresses. Typical methods for finding the suitable dimensions of the embodiment design of a soft robot are the finite element method (FEM) and experimental testing. In the context of FEM analysis, suitable hyperelastic and viscoelastic models for soft materials exist [54]. These models, however, require extensive material characterization. A detailed instruction on how to perform an FEM analysis of different soft actuators can be found in [41].

#### Fabrication

Typical methods for fabricating soft structures are lamination casting (also known as soft lithography) [36], retractable pin casting [36], lost wax casting [36], and rotational casting [55]. In principle, also 3D printing of the soft structure is possible, but currently available printing materials are too brittle compared to casted elastomers [56]. However, there are efforts to use cast elastomers directly in 3D printing [57], which seems a promising alternative to the above-mentioned methods.

### Final realization

In this final stage, the soft robot is fabricated, the control is designed, and the robot is tested.

## Results and discussion

As an example, we use our proposed methodology to design a new climbing soft robot.

### Task definition

In our application, we define the task as follows: “Design a soft machine that is able to walk on inclined surfaces.” For reasons of simplicity, however, the control system of the robot should be outsourced and consist of hard components. Furthermore, we assume the running surfaces to be smooth and free of obstacles.

### Solution search

As already mentioned above, a typical way for finding new solutions in soft robotics is by analyzing natural systems. A suitable natural system for fulfilling the task described above is the gecko [58]. Several examples of gecko-inspired climbing robots exist, including [59, 60]. However, all these robots are made of complex, sensitive components that are most likely to fail in harsh environments. For this reason, we will design a new gecko-inspired soft robot that is resilient to adverse conditions. But in order to do so, we have first to study the actual biological model.

Basically, the gecko consists of 11 limbs: four legs, four feet, a torso, a head, and a tail. The gait pattern of the gecko during wall climbing is illustrated in Fig. 3. We can see that the movement of the torso and legs is symmetrical to the horizontal axis through the center of the torso. Furthermore, only one pair of the diagonally opposite feet is attached to the ground at the same time, and the vertical shift in position is largely achieved by the curvature of the torso. The tail, on the other hand, is used for compensating lateral forces at fast movements.

### Conceptual design

#### Type

Since the gait pattern in Fig. 3 only contains bending movements, we select bending actuators for both the legs and the torso. In order to realize attachment of the soft robot to the ground, we use suction cups for the feet. In our design, a head is not required because the control of the robot is outsourced, and a tail is not used because no high dynamics are expected, and therefore, no compensation of lateral forces is needed.

#### Number

In detail, we require four bending actuators for representing the legs, two bending actuators for representing the torso, and four suction cups for representing the feet of the gecko.

#### Arrangement

A suitable arrangement of the bending actuators and suction cups for realizing the gait pattern from Fig. 3 is illustrated in Fig. 4. Note that the two bending actuators forming the soft robot’s torso share a common non-stretchable part and that this part is extended to the touching ends of the bending actuators that form the legs of the robot.

#### Connection

For reasons of simplicity, in our design, the elements shall be glued together.

### Mechanical modeling

In order to realize the gait pattern from Fig. 3, we developed a mechanical model as illustrated in Fig. 5. The model consists of six bending actuators for the purpose of locomotion and four suction cups for the purpose of adhesion. Under the assumption of a constant curvature [40] of the bending actuators, this model can be described by five degrees of freedom, namely the bending actuators’ curvature angles $\alpha _1$, $\alpha _2$, $\beta _1$, $\beta _2$, and $\gamma$. Note that the two bending actuators representing the soft robot’s torso are described by a common curvature angle $\gamma$. Additionally, we also have four discrete variables, namely the fixation states of the diagonally opposite feet. In the following, we will derive a kinematic model of the soft robot for linear gait by using several constraints.

#### Constant orientation of the attached feet

During the robot’s actuation, the orientation of the attached feet is assumed to be constant. This can be described by the following boundary conditions:

1 $$\begin{aligned} \alpha _i - \frac{\gamma }{2}&= C_{1,i}, \end{aligned}$$

2 $$\begin{aligned} \beta _i + \frac{\gamma }{2}&= C_{2,i}, \end{aligned}$$

where $C_{1,i}$ and $C_{2,i}$ are constants with $i\in \{1,2\}$.

#### Axial symmetry to the horizontal axis through the center of the torso

In order to realize this constraint, the orientations of the right and left feet must be equal:

3 $$\begin{aligned} \alpha _1 = \alpha _2 = \alpha, \end{aligned}$$

4 $$\begin{aligned} \beta _1 = \beta _2 = \beta. \end{aligned}$$

#### Equal orientation of the diagonally opposite feet

This requirement can be formulated as follows:

5 $$\begin{aligned} C_{1,i} = C_{2,i} = C. \end{aligned}$$

#### Nonnegative feet orientation

Since it is technically not possible to obtain negative feet orientation, we assume $\alpha ,\beta \ge 0^\circ$. Furthermore, $\gamma$ is assumed to be $\gamma \in [-90^\circ ,90^\circ ]$. In order to cover the whole $\gamma$ domain and also realize the above equations, the constant *C* has to be chosen as

6 $$\begin{aligned} C = 45^\circ. \end{aligned}$$

With this value, we finally get the following expressions for $\alpha$ and $\beta$:

7 $$\begin{aligned} \alpha (\gamma )&= 45^\circ + \frac{\gamma }{2}, \end{aligned}$$

8 $$\begin{aligned} \beta (\gamma )&= 45^\circ - \frac{\gamma }{2}, \end{aligned}$$

which only depend on $\gamma$. The resulting gait pattern of the robot is shown in Fig. 6. We can see that, for an actuator length of five boxes, one gait cycle of the robot results in a vertical shift in position of seven boxes. The small offset of the lower feet during gait that is given in Figs. 5 and 6 results from the boundary conditions and can thus not be eliminated. However, we assume that this offset is compensated by the high elasticity of the robot.

### Embodiment design

#### Shape

We choose the “fast pneu-net” (fPN) design [51] for the bending actuators of our soft robot because it requires less pressure for the same curvature and can achieve higher bending speeds and forces compared to similar actuator designs. In order to realize a functional integration, the supply tubes are used as the bending actuators’ non-stretchable parts. Furthermore, the bending actuators forming the soft robot’s legs are equipped with side walls for increased stiffness. The partially cut CAD models of the bending actuator designs used in our soft robot are depicted in Fig. 7.

The design of the suction cups is based on the cup design ESV-40-S of Festo [61]. Here, the geometry of the sealing lip has been adopted, and the upper part has been redesigned such that the suction cups can be easily glued to the bending actuators. The partially cut CAD model of the suction cup design used in our soft robot is shown in Fig. 8.

#### Material

The supply tubes shall be made of polyurethane because polyurethane is hardly stretchable but flexible, and the other robot structure shall be made of Elastosil (M 4601) due to this material’s linear pressure–volume behavior in combination with the fPN bending actuator design [51]. The bending actuators and the suction cups shall be actuated pneumatically with air.

#### Surface

In order to avoid a deflection of the soft robot due to gravity, the free bottom surface of the robot is equipped with spherical heads as spacers along the neutral fibers of the bending actuators that have the same height as the sealing lip of the suction cups. Since, compared to the suction cups, the coefficient of friction of the pinheads on different smooth surfaces can be neglected, the pinheads should hardly affect the robot kinematics.

#### Dimension

According to [62], an fPN actuator design with larger height, thinner walls, and higher number of chambers is favorable. In this context, an FEM optimized design has already been introduced in [51]. Therefore, we adopt the dimensions from this work. The thickness and height of the bending actuators’ side walls are chosen intuitively. The dimensions of the upper part of the suction cups are adapted to the bending actuators’ connecting dimensions, and the (outer) diameter of the supply tubes is chosen according to the thickness of the non-stretchable layer. The horizontally cut, exploded CAD model of the embodiment design of our soft robot is shown in Fig. 9. Note that all supply tubes are located inside the robot.

### Final realization

#### Fabrication

Figure 10 shows a partially cut, exploded view of the individual parts of our soft robot. All parts are lamination casted and then glued together by using a thin coat of uncured Elastosil. A photograph of our fabricated robot is depicted in Fig. 11.

#### Control

Due to the different loads on the individual bending actuators during gait as well as manufacturing inaccuracies, the same curvature of the bending actuators does not necessarily correspond to the same pressure level. For this reason, the pressure of each bending actuator is individually controlled by a proportional directional valve, and the valves are connected in parallel to a constant positive pressure source. Since the suction cups have only two states, namely vacuum on and vacuum off, we use direct acting solenoid valves that are parallel connected to a constant negative pressure source for their control. In order to obtain information about the pressure states in the bending actuators, digital pressure sensors are connected to all outputs of the proportional directional valves. A processing unit compares the measured data with the current reference values and then generates the corresponding control signals.

During control, only the extreme positions shown in Fig. 6 are approached (namely $\gamma =90^\circ$ and $\gamma =-90^\circ$), where each $\gamma$ is assigned a set of pressures for all bending actuators that has to be identified experimentally in advance.

#### Experiments

The experiments were performed on an inclined plate made of glass whose inclination angle could be continuously varied. A fixed camera was positioned in front of the plate so that it could optimally capture the running plane. In order to be able to track the gait of the robot, a poster with a chessboard pattern was attached under the plate. The running tests were carried out for different inclination angles $\delta \in \{0^\circ ,10^\circ ,\dots ,90^\circ \}$.

Figure 12a shows the simulation of the soft robot’s gait for one gait cycle. It can be observed that a shift in position of approximately two boxes can be achieved, which corresponds to about 16 cm.

Figure 12b–d shows snapshots of the robot during the first gait cycle for increasing inclination (see also Additional file 1). It can be seen that, for the flat and the moderately inclined plane ($\delta \in \{0^\circ ,\dots ,20^\circ \}$), the gait of the robot is stable and robust and consistent with the simulation. For $\delta \in \{30^\circ ,\dots ,50^\circ \}$, the gait becomes progressively unstable because, during the gripping process, the robot begins to slip increasingly due to a slight twisting of the suction cups. Here, the increasing influence of gravity becomes evident. The motion of the robot is also not completely symmetrical, which causes a slight rotation to the left in the running direction. From $\delta =60^\circ$ onwards, however, no stable gait can be realized.

## Conclusions

In this paper, we introduced a general design methodology for technical systems with an emphasis on soft robotics. The methodology is composed in such a way that the design engineer is guided step by step through the design process. Due to an easy manageability of the design process and a focus on only one aspect at a time, completely new solutions can be created in this way.

The presented approach can be viewed as a framework for a more comprehensive design methodology for soft robotic systems. For example, in the final realization part of the design process, an own methodology for the control design shall be implemented. But also the other aspects should be extended by additional methods and concepts.

The application of our approach was illustrated on the design of a gecko-inspired soft robot that is capable of walking on inclined surfaces. However, our approach does not rely on an existing solution since a unique arrangement of elements can also be realized without a biological or other model [17, 18]. Furthermore, by using our approach, also other known designs can be reproduced and/or optimized, for example, [1, 3, 4, 6, 7, 30, 32, 35, 36, 43, 46, 53, 62].

## Additional file

**Additional file 1.** Performance of the gecko-inspired soft robot at different inclination angles.
